# Pediatric Optic Neuritis: Description of Four Cases and Review of the Literature

**DOI:** 10.3390/children8100855

**Published:** 2021-09-27

**Authors:** Anna Presicci, Maria Serra, Mariaclara Achille, Elvita Caputo, Lucia Margari

**Affiliations:** 1 Department of Neuroscience, Sense Organs and Locomotor System, University Hospital “Policlinico”, Piazza Giulio Cesare 11, 70124 Bari, Italy; 2 Department of Pharmacy-Pharmaceutical Sciences, University of Bari “Aldo Moro”, Piazza Giulio Cesare 11, 70124 Bari, Italy; maria.serra.13@gmail.com; 3 Department of Biomedical Sciences and Human Oncology, University of Bari “Aldo Moro”, Piazza Giulio Cesare 11, 70124 Bari, Italy; mariaclarachille@gmail.com (M.A.); elvitacaputo@gmail.com (E.C.); lucia.margari@uniba.it (L.M.)

**Keywords:** pediatric optic neuritis, acquired demyelinating syndromes, multiple sclerosis, neuromyelitis optica, ADEM-ON, myelin oligodendrocyte glycoprotein

## Abstract

Pediatric optic neuritis (PON) may be a clinically isolated and self-limiting event or may present in the context of underlying neurologic, infective, or systemic disease. PON has a high impact on the quality of life as it may or may not evolve into other acquired demyelinating syndromes (ADSs), such as multiple sclerosis (MS), neuromyelitis optica (NMO), or other syndromes related to the myelin oligodendrocyte glycoprotein IgG antibodies (MOG-IgG). These different PON phenotypes present variable clinical and radiological features, plasma and liquor biomarkers, and prognosis. We describe four pediatric cases presenting clinically with ON, with different etiopathogenetic pictures: one case had a probable infective etiology, while the others were associated with different demyelinating disorders (MS, NMO, syndrome related to MOG-IgG). We discuss the possible evolution of presenting ON in other ADSs, based on recent literature. A careful evaluation of the clinical and investigation findings and the natural course of PON is necessary to define its pathogenic pathway and evolution. Further prolonged follow-up studies are needed to highlight the predictors of PON evolution, its potential sequelae, and the best treatment options.

## 1. Introduction

Pediatric optic neuritis (PON) is the inflammation of the optic nerve, manifesting clinically with acute or subacute visual loss; periorbital pain, dyschromatopsia, and visual field defects frequently co-occur [1,2,3].

The incidence rate is 0.15–0.57 per 100,000 person-years, less than that of adult-onset optic neuritis (AON) estimated at approximately 1–2 individuals per 100,000 per year [2,4]. The majority of PON cases affect postpubertal individuals, with higher female predominance; when prepubertal, an equal incidence in boys and girls is described [1,5].

The optic nerve inflammation may have multiple etiologies (idiopathic, autoimmunity, infection, granulomatous diseases, vasculitides, paraneoplastic disorders, demyelination) [1,4,5,6,7,8]. While the most frequent cause of AON is a demyelinating disorder, PON derives frequently from a parainfective or paravaccination autoimmune process [7,9]. ON is part of acquired demyelinating syndromes (ADSs), a cluster of diseases with different clinical and radiological features, plasma and liquor biomarkers, and prognosis. Their pathogenic pathway is similar in all: an immune-mediated inflammatory process leading to demyelination [10]. The discovery of autoantibodies to the cell membrane water channel aquaporin 4 (AQP4-IgG) and the myelin oligodendrocyte glycoprotein (MOG-IgG) has recently allowed for broadening the differential diagnosis between ADSs [6]. PON has a potentially high impact on the quality of life, as it may or may not evolve into other ADSs, in particular multiple sclerosis (MS), neuromyelitis optica (NMO), or syndromes related to MOG-IgG antibodies [11].

The different phenotypes have variable prognostic features, ranging from the complete absence of sequelae to visual dysfunction and disability in general.

To date, no prospective treatment trials have been performed for PON, so that adult outcomes from the Optic Neuritis Treatment Trial guide the clinical practice [12]. Corticosteroids are the first-line acute treatment of PON, with intravenous 20–30 mg/kg methylprednisolone in 3–5 days (maximum dose of 1 g/day): their anti-inflammatory effects are likely to quicken the visual recovery in the early phase [2,13]. In children, intravenous methylprednisolone should not be used for prolonged periods due to the risk for growth retardation [14]. On the other hand, most children are treated with a slow oral steroid tapering over 2–4 weeks due to the higher relapse risk than adults, although the actual need for a prolonged oral steroid cycle is unknown [1,13]. Plasmapheresis and intravenous immunoglobulins are the preferred second-line therapies [1,13]. When detecting a chronic neuroinflammatory syndrome, specific disease-modifying drugs (DMD) are needed [5].

We describe four cases of PON undertaken by our child neuropsychiatry unit, with different phenotypes, underlying pathologic mechanisms, and evolution. We aimed to provide new information about PON, a rare condition with a relative scarcity of studies, to better understand its heterogeneity in the clinical and investigation findings and prognostic features.

## 2. Case Reports

### 2.1. Investigations

We studied four cases of PON. The diagnostic workup performed on the four patients, after written informed consent obtained from their parents, consisted of the following:

◯ Neurological examination;
◯ Ophthalmological examination, including visual acuity assessment and fundus oculi examination;
◯ Serological examination: laboratory routine blood tests, infectious disease tests, autoantibodies including MOG-IgG and AQP4-IgG;
◯ Cerebrospinal fluid (CSF) examination;
◯ Visual evoked potentials (VEPs);
◯ Computerized visual field (CVF);
◯ Optical coherence tomography (OCT);
◯ Magnetic resonance imaging (MRI).

The results of the diagnostic workup are summarized in Table 1.

### 2.2. Patients

#### 2.2.1. Case 1

**Figure 1:**
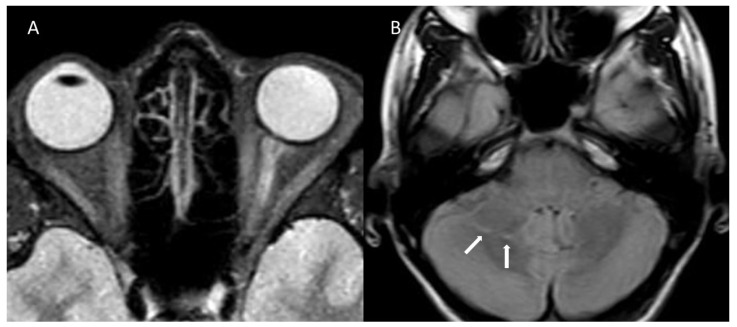
Magnetic resonance imaging scans of case 1. (**A**) Axial short inversion time recovery sequence demonstrates hyperintensity of the left optic nerve. (**B**) Axial fluid-attenuated inversion recovery scan of the brain shows two millimetric hyperintense cerebellar foci (arrows).

The first case is a 17-year-old girl with a family history of MS, hemicrania, and general anxiety disorder, without perinatal risk factors. At age 16, she presented with hypophagia, significant weight loss, asthenia, paraesthesia of both hands, sleep–wake cycle anomalies, and amenorrhea. These symptoms were followed, after a few weeks, by an acute onset of blurry vision in the left eye, scotomas, associated with supraorbital pain exacerbated by eye movements, progressively worsening within hours. She was diagnosed with unilateral, postinfective ON, likely secondary to Epstein–Barr virus (EBV) infection. MRI scans are shown in Figure 1. The patient was treated with high intravenous doses of methylprednisolone, with a complete recovery of visual acuity. The mild papillitis in the left eye persisted for five months after the ON onset. No other demyelinating events occurred in the following year of follow-up.

#### 2.2.2. Case 2

**Figure 2:**
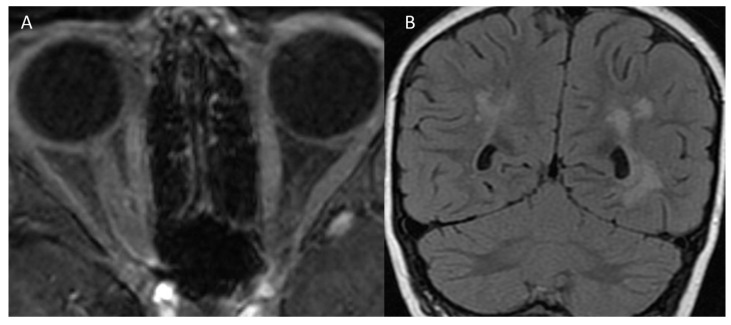
Magnetic resonance imaging scans of case 2. (**A**) Axial T1 post-gadolinium water selective orbital scan demonstrates enhancement of the right optic nerve. (**B**) Coronal T2 weighted scan shows multiple bilateral hyperintense white matter lesions in subcortical and periventricular areas of the brain.

The second case is an 11-year-old female with a family history of hematologic and neoplastic conditions, without perinatal risk factors. At age 3, she presented with an episode of acute disseminated encephalomyelitis (ADEM). Plasmapheresis and steroids were prompted, with a full recovery. At age 7, she showed an acute onset of periorbital pain, blurriness, and loss of visual acuity in the right eye. One month before the ON, she presented a generalized critical episode. MRI scans showed the inflammation of the right optic nerve with neuroradiological outcomes of the previous ADEM (Figure 2)**.** She was diagnosed with ADEM followed by ON (ADEM-ON), MOG-IgG seropositive. The patient was treated with intravenous methylprednisolone, with a gradual, partial recovery of visual acuity. In the following three years, she presented two more generalized critical episodes. Antiepileptic therapy was proposed, but the family refused.

#### 2.2.3. Case 3

**Figure 3:**
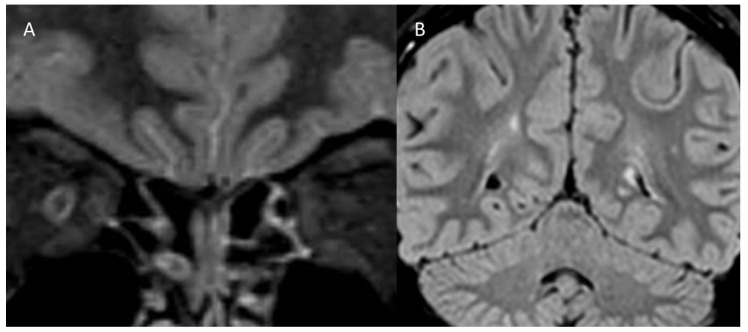
Magnetic resonance imaging scans of case 3. (**A**) Coronal short inversion time recovery sequence shows hyperintensity of the right optic nerve. (**B**) Coronal fluid-attenuated inversion recovery section of the brain demonstrates multiple bilateral hyperintense periventricular and juxtacortical white matter lesions.

The third case is a 17-year-old girl with a family history of intellectual disability and headache. She was born from twin birth, with an occurrence of threatened miscarriage in the first trimester. At age 7, she was diagnosed with a learning disorder and separation anxiety disorder. At age 17, she suffered from an acute visual loss in the right eye, blurred vision, supraorbital pain, and dyschromatopsia. One month before, she presented episodes of frontal constrictive headache, with cold sweating and urinary urgency, following prolonged sun exposure. She was diagnosed with MS because the acute unilateral ON was associated with MRI signs of spatial and temporal dissemination (Figure 3). The patient was treated with intravenous methylprednisolone, followed by oral tapering, with a complete recovery of visual acuity. Three months later, MRI scans showed a new demyelinating lesion; specific DMD treatment was started.

#### 2.2.4. Case 4

**Figure 4:**
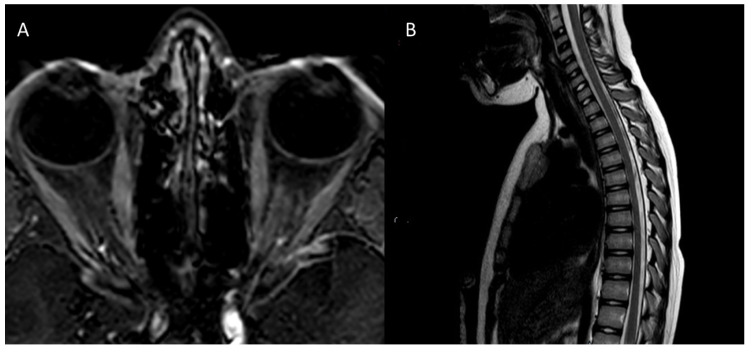
Magnetic resonance imaging scans of case 4. (**A**) Axial T1 post-gadolinium water selective orbital scan demonstrates a mild enhancement of the left optic nerve lasting 45 days after the onset. (**B**) Sagittal spinal cord T2 weighted scan shows the absence of medullary demyelinating lesions, with transverse myelitis being more common as a recurrence than an onset symptom of neuromyelitis optica.

The fourth case is a 10-year-old female with a family history of factor V Leiden mutation, psoriasis, and Raynaud disease, without perinatal risk factors. At age 8, she presented with a severe loss of visual acuity in both eyes, preceded by head trauma. She was diagnosed with NMO, AQP4-IgG seropositive. The optic nerve inflammation was bilateral but more pronounced in the left side, where MRI signs of ON lasted for more than one month after the onset. The spinal cord was free from demyelinating lesions (Figure 4). She was treated with intravenous methylprednisolone followed by oral steroid tapering, with poor visual recovery. The patient underwent plasmapheresis. After 13 sessions, almost complete visual recovery was achieved in the right eye, while the deficit persisted in the left eye.

## 3. Discussion

We discuss four cases referring to the current literature on PON, evaluating risk factors for the development of other ADSs, such as MS and NMO, considering the crucial role of MOG-IgG and AQP4-IgG.

The cases are all female, and two of them are in postpubertal age, in accordance with the epidemiologic data of the literature [1,5].

ON is frequently limited to an isolated and self-limiting event, although the diagnosis of a single isolated optic neuritis (SION) cannot be made if not after a period of follow-up, making the definition of its prevalence more difficult [6]. Isolated PON was described in the 17% of 53 pediatric patients studied along a mean follow-up period of 25 months, with no underlying diseases identified [15]. In our sample, case 1 was diagnosed with unilateral, postinfective ON, likely secondary to EBV infection. ON is a typical EBV-related neurological disease [16]. The coexistence of positive anti-viral capsid antigen (VCA) and anti-Epstein–Barr nuclear antigen (EBNA) EBV IgG and doubtfully positive VCA EBV IgM is interpretable with a recent EBV infection or with a viral reactivation, likely responsible for the physical symptoms preceding the ON. The single liquor OCB, not detected in serum, could represent the intrathecal anti-EBV IgG synthesis responsible for the clinical manifestations. The detection of seven plasmatic OCBs could mean transient paraproteinemia, which can occur after infection and persist for a few months. If not infectious—because of the doubtful positivity for VCA EBV IgM—case 1 ON could be a SION, needing confirmation after a follow-up period [6].

ON may not be isolated and may evolve into other ADSs [6].

In our sample, case 2 presented ADEM-like early-onset ADS, with a multiphasic course (relapse with ON) and high titer seropositivity for MOG-IgG, diagnosable as ADEM-ON, a relatively new nosologic entity with monophasic or recurrent ON following a previous ADEM [17]. The detection of MOG-IgG strongly underpins ADEM-ON diagnosis, confirming this entity as part of MOG-IgG-related disorders [17]. ON is a common onset in MOG-IgG-related disorders, mainly in individuals older than nine years, while younger children MOG-IgG seropositive are more likely to present with ADEM [3].

ON represents the onset symptom of pediatric MS in 20–25% of cases [1,4,18]. The PON evolution towards MS in the pediatric population has not yet been the subject of randomized studies. Available data show a risk of MS development variable from 4 to 37% [5,9,19,20]. The main factor associated with an increased risk for MS is detecting one or more neuroradiological lesions suggestive of MS, as in adults. Other factors were also identified, such as the age greater than ten years and the presence of oligoclonal bands (OCBs) in CSF [21,22]. Low titers of MOG-IgG have been detected in the early stage of MS; otherwise, elevated levels of MOG-IgG are usually not associated with MS [23]. Case 3 met the diagnostic criteria for pediatric MS, given the presence of a typical clinically isolated syndrome (a unilateral ON, extended to small portions of the nerve and associated with moderate pain) and neuroradiological evidence of spatial and temporal dissemination [24,25,26]. Since this is a pediatric MS, a relapsing–remitting course is likely, as it occurs in 98% of onset before 18 years [27]. The risk of other demyelinating events is higher in the first two or three years after the onset [2,3]. Despite a slower progression rate than in adult MS, the earlier onset makes an irreversible disability occur earlier [28]. The presence of a new encephalic lesion at the third month MRI follow-up showed disease activity. Specific therapy with DMD was performed promptly to reduce long-term disability [2]. A poor visual outcome is expected to occur, with probable visual field deficits [29]. For cases 1 and 2, we considered the risk factors for the development of MS. In case 1, we should not exclude the possible evolution towards MS, because of the postpubertal onset of the ON, the presence of millimetric T2 hyperintense foci in the cerebellum, and the detection of a single liquor OCB, which is not diagnostic but a possible early sign [30]. Furthermore, it is important to consider a potential contribution of EBV infection to the pathogenesis of MS by potentiating the inflammatory mechanism [16]. In case 2, the clinical features (age less than 10) and the investigation findings (elevated levels of MOG-IgG, absence of OCBs in CSF examination, no demyelinating brain lesions) do not support evolution in MS, even if they do not categorically exclude it.

ON is a symptom of onset in more than half of NMO cases: it is often bilateral and associated with positive AQP4-IgG, which are highly specific for NMO and are now considered a major diagnostic criterion [31]. A group of NMO patients can be seronegative for AQP4-IgG, falling under the designation of NMO spectrum disease (NMO-SD) [3]. A subset of these subjects are positive for MOG-IgG [3,32]. The evolution of ON towards NMO varies from 1 to 7% [22]. Radiological features of NMO-ON are long extension, chiasmatic and optic tract involvement, nuanced edema, and nerve tortuosity [6]. However, the most important information for evolution towards NMO is the sensitivity for APQ4-IgG [1,33]. In such cases, a severe visual impairment is likely to occur. NMO associated with MOG-IgG has an early onset and is associated with long extension ON without involvement of the spinal cord and severe papillae edema [6]. In a prognostic sight, relapses can easily occur in NMO patients, mainly if AQP4-IgG seropositive; while the onset affects the optic nerves more commonly, transverse myelitis is frequent in recurrences [3,34]. After plasmapheresis, a specific DMD is recommended for the prevention of recurrences [2]. Case 4 was diagnosed with AQP4-IgG seropositive NMO, with a bilateral ON as the clinical onset. NMO forms of PON are particularly severe, maybe due to the direct astrocytopathy and neural necrosis caused by AQP4-IgG and the frequent bilateral involvement [2]. A thinning of the retinal ganglion cell layer was observed through the OCT in patient 4, as described in the literature about NMO-ON, correlating with the severity of visual dysfunction [4]. Thirteen sessions of plasmapheresis were performed, as preferred early second-line therapy after intravenous methylprednisolone, with the aim of a better visual outcome [3]. Good visual recovery was achieved in the right eye while a severe deficit persisted in the left one, signaling the severity of this condition. About the risk of developing NMO, it is very scarce for case 1, in the absence of its clinical, laboratory, and neuroradiological features. In case 2 a possible evolution into an AQP4-IgG seronegative and MOG-IgG seropositive NMO cannot be ruled out, even if the clinical phenotype does not match the current diagnostic criteria for NMO.

All cases presented clinically with the typical ON signs and symptoms, but the evolution and prognosis were different. While the etiopathogenesis of PON seems clearer for cases 1, 3, and 4, for case 2 it remains uncertain. In particular, at the moment, patient 2 can be included in the spectrum of multiphasic clinical syndromes related to MOG-IgG, considered by some authors as a distinct clinical entity, on the border between MS and NMO [6,10,35]. In this case, a persistently high titer of MOG-IgG could be a predicting factor of further recurrences, based on recent data [35].

The recent literature helped to increase the knowledge on the evolution of PON, a rare condition with a relative scarcity of studies and without well-defined prognosis and standardized follow-up paths. This study could provide new information about PON, its heterogeneity in the clinical and investigation findings, the risk factors for other ADSs, and prognosis.

## 4. Conclusions

Many different conditions can present clinically with ON, but their etiopathogenetic processes, response to treatment, visual outcome, and relapsing risk may vary substantially. The analysis of the sample examined in this study gives information that may help to understand the high heterogeneity of PON and the necessary evaluation of its natural course to define its pathogenic pathway and evolution. Further prolonged follow-up studies are needed to highlight the predictors of PON evolution, its potential sequelae, and the best treatment options.

## Figures and Tables

**Table 1:** Diagnostic workup results of the four cases.

	Case 1	Case 2	Case 3	Case 4
Neurological examination	Weakness of the right upper limb with pronation of the hand	Right pyramidal signs	Left pyramidal signs	Hyporeflexia, Horizontal nystagmus
Ophthalmological examination	LE: NV 8/10, mild papillitis	RE: CV 1/50, significant papillitis	RE: NV 3/10, no signs of papillitis	RE: NV 1/100, mild papillitis LE: NV hand motion
		Severe dyschromatopsia		
Laboratory findings	VCA and EBNA EBV IgG: positive VCA EBV IgM: doubtful positive CSF: 1 OCB absent in the serum; 7 serum OCBs	MOG-IgG: high titer CSF: pleocytosis, intrathecal IgA synthesis	MOG-IgG: low titer CSF: 7 OCBs, absent in the serum	AQP4-IgG: high titer
CVF	LE: reduced central and upper sensitivity	RE: central scotoma	RE: small central scotoma	NA
VEP	LE: increased latency	RE: increased latency	RE: amplitude reduction	Bilateral increased latency and amplitude reduction
OCT	LE: increased retinal nerve fiber layer	NA	Normal findings	RE: reduced temporal ganglion cell layer LE: NA
MRI	Left optic nerve: hyperintensity (T2) Two millimetric T2 hyperintense cerebellar foci	Right optic nerve: hyperintensity (STIR), contrast enhancement ADEM outcomes	Right optic nerve: small hyperintensity (T2) Multiple juxtacortical, infratentorial, and periventricular demyelinating lesions; four lesions with contrast enhancement	Right and left optic nerves: retrobulbar bilateral swelling, contrast enhancement

NV: natural vision. CV: correct vision. RE: right eye. LE: left eye. MOG-IgG: autoantibodies to the myelin oligodendrocyte glycoprotein. AQP4-IgG: autoantibodies to the cell membrane water channel aquaporin 4. CSF: cerebrospinal fluid. CVF: computerized visual field. VEP: visual evoked potential. OCT: optical coherence tomography. MRI: magnetic resonance imaging.

## Data Availability

The data presented in this study are available within the article.
